# Post-SARS-CoV-2 vaccination venous sinus thrombosis: a literature review of 308 cases

**DOI:** 10.1186/s41983-021-00431-z

**Published:** 2021-12-20

**Authors:** Josef Finsterer, Sinda Zarrouk

**Affiliations:** 1 Klinik Landstrasse, Messerli Institute, Postfach 20, 1180 Vienna, Austria; 2 University of Tunis El Manar and Genomics Platform, Pasteur Institute of Tunis, Tunis, Tunisia

**Keywords:** SARS-CoV-2, Coronavirus, COVID-19, Thrombosis, Hypercoagulability, Covid vaccines

## Abstract

There is accumulating evidence that SARS-CoV-2 vaccinations can be complicated by venous sinus thrombosis (VST). This review aimed at summarising and discussing previous and recent advances regarding the diagnosis, pathogenesis, treatment, and outcome of post-SARS-CoV-2 vaccination VST. At least 308 patients with post-SARS-CoV-2 vaccination VST have been reported as per the end of July 2021. Ages among these 308 patients ranged between 22 and 81 years, 69 were male and 197 were female. Post-SARS-CoV-2 vaccination VST most commonly occurred with the ChAdOx1-S vaccine followed by the BNT126b2 vaccine. In the vast majority of cases, VST occurred after the first dose. Only in six patients did VST occur after the second dose. Latency between vaccination and onset of VST ranged between 0 and 24 days. Regarding treatment, most patients received heparin followed by oral anticoagulants. Seven patients received IVIGs and six patients received steroids because of concomitant vaccine-induced immune thrombotic thrombocytopenia. Complete recovery was reported in 5 patients. Partial recovery was reported in 9 patients. Eight patients were alive or discharged. Sixty-two patients died. The outcome was not specified in the remainder. In conclusion, SARS-CoV-2 vaccinations can be complicated by VST. There is female preponderance and the outcome is frequently poor.

## Main text

In SARS-CoV-2 infected, the increased risk of thrombosis is explained by virus-induced endotheliopathy or coagulopathy [1]. Invasion of the virus into endothelial cells causes their dysfunction or disruption. Dysfunction of endothelial cells leads to reduced action of thrombomodulin, which reduces coagulation in normal quantities. Disruption of endothelial cells leads to release of the plasminogen activator inhibitor-1 (PAI-1), which blocks the action of proteins that cause clot lysis and consecutively reduce the possibility of bleeding. Thus, increased levels of PAI-1 in COVID-19 enhance coagulopathy. Thrombosis in COVID-19 can be also explained by the increased Von Willebrand factor, which enhances platelet adherence and stabilises blood clots. Another mechanism explaining thrombosis in COVID-19 is the increased release of inorganic phosphate residues from thrombocytes, stimulating the release of factor V and factor XI. Since cytokines and chemokines, which are excessively released in COVID-19 patients, activate macrophages, the increased macrophage count also increases the risk of clot formation [1].

Venous sinus thrombosis (VST) has been repeatedly reported as a complication of SARS-CoV-2 infections [2, 3]. Additionally, there is accumulating evidence that VST can be also a complication of SARS-CoV-2 vaccinations [4–6]. Upon anticoagulation with heparin or oral anticoagulants, respectively, symptoms and signs of VST usually resolve, but a number of fatalities have been reported as well. Particularly, in case VST is associated with thrombocytopenia, the outcome can be fatal or unfavourable (Table 1). Though the pathophysiological mechanism underlying the development of thrombosis after SARS-CoV-2 vaccinations has not been fully elucidated, there are indications that the immune response towards the vaccine creates a state of hypercoagulability similar to hypercoagulability in SARS-CoV-2 infections [7], but hypocoagulability has been also reported [8]. This review aimed at summarising and discussing previous and recent advances regarding the diagnosis, pathogenesis, treatment, and outcome of post-SARS-CoV-2 vaccination venous sinus thrombosis.

**Table 1:** Patients with post-SARS-CoV-2 vaccination VST reported in the literature

Age	Sex	Dose	Company	locSVT	Latency (days)	Therapy	Outcome	References
53	f	1st	A	Cavernous	11	Intubation, platelets	Death	[9]
55	m	1st	A	Cavernous	8	Steroids, argatroban	Death	[9]
30*	f	1st	A	Transverse, sigmoid	7	Argatroban, IVIG	PR	[10]
30	f	1st	A	nr	9	Tinzaparin, rivaroxaban	PR	[11]
27*	m	1st	A	nr	2	Dabigatran, IVIG	Death	[12]
54*	m	nr	A	nr	14	Danaparoid, IVIG, DOAC	Alive	[13]
49	m	1st	P	Transverse, sigmoid	16	Clexane, apixaban	Alive	[14]
69	f	1st	A	Sigmoid, SSS	11	nr	Death	[5]
32	m	1st	A	SSS	9	None	Death	[14]
25*	m	1st	A	SSS	6	Heparin, platelets, IVIG, steroids	Death	[14]
22	f	1st	A	SSS, transverse	4	Enoxaparin, dabigatran	PR	[15]
46	f	1st	A	SSS, sigmoid, transverse	8	Danaparoid, dabigatran	PR	[15]
36	f	1st	A	SSS, straight	7	Enoxaparin, dabigatran	CR	[15]
50	m	1st	A	Transverse, sigmoid	7	Craniotomy	Death	[16]
61	f	nr	A	nr	14	Enoxaparin	Discharged	[17]
40	m	1st	A	nr	14	Enoxaparin, apixaban	Discharged	[17]
50	m	1st	A	SSS, sigmoid	7	Enoxaparin, craniotomy	Death	[18]
42	f	1st	A	SSS, straight sigmoid	0	Enoxaparin, craniotomy	Coma	[18]
55	f	2nd	P	Straight, sigmoid	1	Enoxaparin	Death	[18]
32	f	1st	A	Straight, sigmoid	1	Fondaparinux, steroids	Death	[18]
35	f	1st	A	SSS, straight sigmoid	6	Enoxaparin, steroids, PE	Coma	[18]
51	f	1st	A	Straight, sigmoid	10	Ventriculostomy	Death	[18]
64	m	1st	A	SSS, straight, sigmoid	4	Enoxaparin	CR	[18]
40	f	1st	A	SSS, straight sigmoid	5	Fondaparinux	PR	[18]
49	f	1st	A	straight, sigmoid	11	Enoxaparin	PR	[18]
54	f	1st	A	SSS, Galen	2	Enoxaparin, steroids	Death	[18]
55	f	1st	A	Jugular	6	Fondaparinux craniotomy	Death	[18]
54	f	nr	A	Labbe, Galen	12	Intubation	Death	[20]
39	f	1st	A	Sigmoid, transverse	6	Danaparoid dabigatran, IVIG	CR	[20]
24*	f	1st	A	Cortical	8	Argatroban, steroids, IVIG	CR	[21]
32	f	1st	A	nr	11	nr	Death	[22]
29*	m	1st	A	Sigmoid, transverse	9	IVIG, agratroban	Recovery	[23]
>40	nr	nr	J	Transverse, sigmoid	6	nr	ND	[23]
18-39	nr	nr	J	Transverse, sigmoid	9	nr	Discharged	[23]
18-39	nr	nr	J	SSS, straight,	8	nr	ND	[23]
18-39	nr	nr	J	Transverse, sigmoid	8	nr	Discharged	[23]
18-39	nr	nr	J	Transverse, sigmoid	6	nr	ND	[23]
>40	nr	nr	J	Transverse, straight	13	nr	ND	[23]
18-39	nr	nr	J	SSS, straight, transverse	15	nr	ND	[23]
18-39	nr	nr	J	Transverse, sigmoid	10	nr	Discharged	[23]
>40	nr	nr	J	SSS, cortical	7	nr	ND	[23]
18-39	nr	nr	J	Transverse, SSS, sigmoid	7	nr	ND	[23]
18-39	nr	nr	J	Transverse, sigmoid	11	nr	ND	[23]
>40	nr	nr	J	Transverse, sigmoid	6	nr	Discharged	[24]
51	m	1st	C	SSS, transverse	6	Heparin, warfarin	PR	[25]
40	f	1st	J	nr	5	Bivalirudin	CR	[26]
36	f	1st	A	SSS	14	Enoxaparin	Death	[27]
25	f	1st	A	Cortical	12	nr	nr	[28]
50*	m	1st	A	Transverse, sigmoid	14	Desirudin	PR	[29]
30*	f	1st	A	None	8	Tinzaparin	PR	[11]
ϴ46.7 (*n* = 45)	35f	1st (42)	A (*n* = 37)	nr	nr	nr	nr	[30]
		2nd (3)	P (*n* = 8)					
32-81 (*n* = 213)	138 f 46 m	1st (except 2)	P (*n* = 25) M (*n* = 1) A (*n* = 187)	nr	2-21d	nr	Death (*n* = 46)	[31]

A: ChAdOx1-S, C: *ChAdOx1-S from India,* CR: complete recovery, DOAC: direct oral anticoagulant, locSVT: location of venous sinus thrombosis, ip: in preparation, IVIG: intravenous immunoglobulins, J: JNJ-78436735, M: mRNA-1273, ND: not discharged, nr: not reported, P: BNT126b2, PE: plasma exchange, PR: partial recovery at discharge or late follow-up (patient had not reached his pre-morbid condition), SSS: superior sagittal sinus, *: associated with immune thrombocytopenia

A literature search was conducted in the databases PubMed and Google Scholar using the search terms “SARS-CoV-2 vaccination”, “mRNA based vaccine”, “vector-based vaccine” combined with “side effect”, “adverse reaction”, “thrombosis”, “venous sinus thrombosis”, and “cerebral veins”. Additionally, reference lists of available articles were checked for further appropriate references. Included were articles which provided detailed information about individual patients experiencing a VST time-linked to the first or second dose of a SARS-CoV-2 vaccine. Excluded were abstracts and proceedings. Four unpublished cases were not included. Only articles written in English were considered.

At least 308 patients with post-SARS-CoV-2 vaccination VST have been reported as per the end of July 2021 (Table 1) [5, 9–31]^.^ Age of these 308 patients ranged between 22 and 81 years. Gender was reported in 270 patients and was male in 69 and female in 197 patients. VST was most frequently reported after application of the ChAdOx1-S vaccine followed by the BNT126b2 and the JNJ-78436735 vaccines. In the vast majority of cases, VST occurred already after the first dose of the vaccine. Only in six patients did VST occur after the second dose. Latency between vaccination and onset of VST was reported in 263 patients and ranged between 0 and 24 days. Treatment of VST was reported in only 35 patients. Most patients received heparin followed by oral anticoagulants. Seven patients received IVIGs and six patients steroids because of concomitant vaccine-induced immune thrombotic thrombocytopenia [27]. Complete recovery was reported in only 5 patients. Partial recovery was reported in 9 patients. The outcome was reported as “alive” in two patients, as “coma” in one patient, and as “discharged” in 6 patients. Sixty-two patients died (Table 1). The outcome was not specified in the remaining patients.

This review shows that VST is indeed a potential complication of SARS-CoV-2 vaccinations usually after the first dose, that VST as a complication of SARS-CoV-2 vaccinations occurs at any age and with female preponderance, that the latency between vaccination and onset of VST ranges between 0 and 24 days, and that VST can be associated with immune thrombocytopenia, whether VST and SARS-CoV-2 vaccinations are causally related remains speculative, but there are several arguments for and against causality. Arguments in favour of a causal relation are (1) that VST occurs time-linked to the vaccination within 24 days after the first or second jab; (2) that thrombotic events have been reported as a complication of SARS-CoV-2 vaccinations in several studies; (3) that SARS-CoV-2 vaccinations can be complicated by hypercoagulability [7], and (4) that post-SARS-CoV-2 vaccination VST has been reported in over 300 patients as per the end of June 2021 (Table 1). Hypercoagulability after SARS-CoV-2 vaccinations can be explained by direct activation of platelets, enhancing coagulation, by indirect activation of endothelial cells, shifting endothelium from an anti-thrombotic to a pro-thrombotic state, and by direct activation of complement pathways, promoting thrombin generation [32]. A pro-coagulant state after SARS-CoV-2 vaccinations could be also explained by increased blood viscosity due to activation of the coagulation pathways. Furthermore, complement consumption, which can be found during the immune response against the virus or the vaccine, may rapidly enhance monocyte tissue factor pro-coagulant activity and thus immuno-thrombosis [33]. Hypercoagulability may not only develop in patients with comorbidities, but also in previously healthy subjects. Arguments against a causal relation are that some studies did not find hypercoagulability after SARS-CoV-2 vaccinations [9], that millions of subjects have been vaccinated without experiencing a thrombotic event, and that the overall incidence/prevalence of VST has apparently not increased when comparing non-COVID and vaccination periods However, mild or small VST may go undiagnosed and undetected as most patients with post-vaccination headache or other complaints do not undergo cerebral imaging with contrast medium and determination of the D-dimer why the true prevalence of post-vaccination VST is expected to be higher than anticipated.

The pathophysiological background of VST following immunisation against the SARS-CoV-2 virus is poorly understood. A possible mechanism could be that the vaccine triggers the formation of anti-platelet factor-4 antibodies, which induce thrombocytopenia with dysfunctional thrombocytes [34]. It is also conceivable that the immunogenic component of the vaccine triggers the formation of antibodies which in turn cross-react with membrane-bound or soluble proteins involved in thrombus formation. Thus, an antibody cross-reaction may be the most likely causal link between VST and immunisation to SARS-CoV-2 [35]. The development of VST within 24 h after the vaccination [10] could be explained by a subclinical pre-morbid hypercoagulable state. The reason why VST occurred already after the first jab remains elusive, but it can be speculated that the immune reaction against the vaccine starts as soon as the antigen is presented. The immune system will not wait until the second jab. The reason why VST was reported only in association with five brands is unknown, but it can be speculated that the more exotic vaccines are not widely approved in countries where publication activity is high.

Whether patients with post-vaccination VST and vaccine-induced thrombotic thrombocytopenia should not receive heparin is unsolved, but there is increasing evidence that heparin should be avoided in these patients [36]. Some studies even recommend the administration of steroids, intravenous immunoglobulins (IVIGs) and antidotes against the platelet factor-4 [37].

A limitation of the review is that insufficient individual data were reported in several articles, particularly the one by See and colleagues (Table 1) [24]. The article by Bikdeli and colleagues was not included in the evaluation as no individual data were provided for the 77 cases with post-vaccination VST reported by the Medicines and Healthcare Products Regulatory Agency (MHPRA). For the same reason, the six cases reported by the Centers for Disease Control and Prevention (CDC) were excluded. The three cases reported by the Society of Vascular and Interventional Neurology were excluded because patients experienced VST after a SARS-CoV-2 infection but not after the vaccination [38]. Another limitation of the study is that almost all studies included did not test the included patients prospectively for the pre-morbid liability of thrombosis.

## Conclusions

SARS-CoV-2 vaccinations can be complicated by VST. VST occurs at any age, predominantly after the first dose, predominatly in females, and with any of vaccine brands. Currently, more arguments in favour than against a causal relation between SARS-CoV-2 vaccination and VST can be raised. As post-vaccination VST may have a fatal outcome, treating physicians need to stay vigilant not to overlook VST and to start treatment in due time.

## Data Availability

Not applicable.

## Abbreviations

CDC: Centers for Disease Control and Prevention
COVID-19: Coronavirus diseases 2019
CSF: Cerebro-spinal fluid
CT: Computed tomography
IVIGs: Intravenous immunoglobulins
PAI-1: Plasminogen activator inhibitor-1
PF: Platelet factor
SARS-CoV-2: Severe, acute, respiratory syndrome-coronavirus-2
VST: Venous sinus thrombosis
